# Association of Preoperative Lymphocyte-to-Monocyte Ratio with Failure to Achieve Textbook Outcome After Pancreatoduodenectomy for Pancreatic and Periampullary Cancer

**DOI:** 10.3390/cancers18183055

**Published:** 2026-09-21

**Authors:** Heleen Driessens, Olav DJ Zwerver, Maarten W. Nijkamp, Alain R. Viddeleer, Joost M. Klaase

**Affiliations:** 1 Department of Surgery, Division of Hepato-Pancreato-Biliary Surgery and Liver Transplantation, University Medical Center Groningen, University of Groningen, 9713 GZ Groningen, The Netherlands; h.driessens@umcg.nl (H.D.);; 2 Department of Radiology, Medical Imaging Center, University Medical Center Groningen, University of Groningen, 9700 RB Groningen, The Netherlands

**Keywords:** pancreatoduodenectomy, pancreatic cancer, lymphocyte-to-monocyte ratio, textbook outcome, preoperative optimization, prehabilitation

## Abstract

Introduction: Pancreatoduodenectomy (PD) is associated with substantial morbidity, underscoring the need for preoperative risk stratification. This study evaluated whether objective, partially modifiable, preoperative host factors, including aerobic fitness, body composition, and immunonutritional biomarkers, are associated with failure to achieve textbook outcome (TO) after PD. Materials and Methods: This retrospective cohort study included patients with malignant pancreatic or periampullary tumors, who performed a routine cardiopulmonary exercise test (CPET) and underwent PD. Body composition was analyzed with CT. Immunonutritional biomarkers included lymphocyte-to-monocyte ratio (LMR), platelet-to-lymphocyte ratio (PLR), neutrophil-to-lymphocyte ratio (NLR), prognostic nutritional index (PNI) and CRP-to-albumin ratio (CAR). TO was defined as the absence of postoperative pancreatic fistula (POPF), bile leak, post-pancreatectomy hemorrhage, major complications, 30-day readmission and 30-day mortality. Results: In total, 105 patients were included, of whom 47 patients (44.8%) failed to achieve TO. Compared with the TO group, non-TO patients were older (70.2 ± 7.8 vs. 66.8 ± 9.0 years), had higher PLR (194.9 [147.3–274.1] vs. 162.8 [105.5–224.6], *p* = 0.016) and lower LMR (2.1 [1.4–3.2] vs. 2.9 [1.9–4.2], *p* = 0.005). Aerobic fitness, body composition, and other biomarkers did not differ significantly. In multivariable analysis, LMR (OR 0.588, 95%-CI 0.406–0.853, *p* = 0.005) and risk of POPF (OR 3.410 for intermediate risk (95%-CI 1.184–9.817, *p* = 0.023) and OR 3.877 for high risk (95%-CI 1.310–11.473, *p* = 0.014) compared to low risk) were independently associated with failure to achieve TO. Conclusions: Lower LMR adjusted for higher fistula risk score independently predicts failure to achieve TO after PD. Preoperative LMR may serve as a simple, objective predictor and a potential target for preoperative optimization.

## Simple Summary

Patients undergoing a major operation of the pancreas or surrounding tissue are known to have frequent complications such as prolonged hospital length of stay and re-operation. One way to describe this is to see if patients have ideal recovery after surgery without complications. It could be wise to know beforehand which patients are at risk for these complications. In this study, based on already collected data of 105 patients, several measurable factors known to influence surgical outcomes (such as fitness, body composition and nutritional status) before surgery were assessed to estimate the chance of complications around the operation in the pancreatic region. This study found that one blood marker, lymphocyte-to-monocyte ratio, was related to not having ideal recovery after surgery. Patients with lower value of this marker were less likely to reach an optimal outcome after surgery. This suggests that this routine blood test may help in assessing risk before surgery and can be used as a potential target for preoperative optimization aiming to reduce complications.

## 1. Introduction

Pancreatic surgery remains the cornerstone of curative treatment for pancreatic and periampullary malignancies [1]. However, pancreatic surgery, particularly pancreatoduodenectomy (PD), is a complex and high-risk procedure, associated with major postoperative complications in up to 30% of cases [2]. While improvements such as centralization of care, enhanced recovery after surgery (ERAS) protocols, and minimally invasive techniques have been widely adopted, postoperative morbidity has remained largely unchanged [3]. This highlights the need for accurate preoperative risk stratification to identify high-risk patients [4,5]. Many established risk-models focus on postoperative pancreatic fistula (POPF) and make use of intra- or postoperative data such as pancreatic duct diameter, pancreatic texture or pathology [6]. However, such risk-models cannot be used in the preoperative context for risk stratification and shared decision-making. A risk model consisting of objective host factors, which are available in the preoperative period, could be a valuable adjunct or alternative, and could be even more worthwhile when potentially modifiable.

To comprehensively assess surgical success, composite endpoints such as the textbook outcome (TO) have been introduced [7]. Defined by van Roessel et al., TO represents the ideal postoperative course, including the absence of major complications, pancreatic fistula, post-pancreatectomy hemorrhage, bile leak, 30-day mortality, and readmission [8]. TO is increasingly used as a patient-centered quality indicator in pancreatic surgery and provides a holistic outcome for evaluating predictors [9]. In pancreatic surgery, a recent meta-analysis identified five risk factors for not achieving a TO: preoperative biliary drainage, smaller tumor size, soft pancreatic texture, small pancreatic duct diameter and intraoperative blood loss [10]. However, in this analysis preoperative host factors were underrepresented.

Previous studies have demonstrated that poor aerobic fitness, as measured by cardiopulmonary exercise testing (CPET), is associated with increased postoperative morbidity in major abdominal surgery [11,12]. We recently confirmed this in patients who underwent pancreatoduodenectomy at our center, demonstrating that low preoperative aerobic fitness was associated with an increased rate of gastroparesis and prolonged hospital stay [13]. Body composition, specifically low skeletal muscle mass and density, determined via preoperative CT at the third lumbar vertebral level (L3), has also been associated with postoperative complications, hospital stay and overall survival in pancreatic surgery [14,15,16]. In addition to aerobic capacity and body composition, immune and nutritional status are increasingly recognized as predictors of surgical outcomes and long-term survival [17,18,19,20]. Immunonutritional biomarkers and nutritional status have already shown a potential association with short-term postoperative complications in pancreatic surgery [21,22,23].

To date, no studies have looked for an association between the combination of aerobic fitness, body composition and immunonutritional status with TO in pancreatic surgery. Therefore, the aim of this study is to investigate whether objective, and partially modifiable, preoperative host factors, including aerobic fitness, body composition, and immunonutritional biomarkers, are associated with failure to achieve TO following PD. Identification of these host factors creates opportunities for preoperative optimization of these factors and might improve the chances of achieving TO.

## 2. Methods

### 2.1. Study Design

This observational retrospective cohort study was conducted between January 2019 and December 2024 at the University Medical Center Groningen, The Netherlands. This study is a subgroup analysis of the FRAIL study, whereby a preoperative prehabilitation care pathway was implemented for all patients scheduled to undergo hepato-pancreato-biliary (HPB) surgery [24]. The FRAIL study was approved by the Institutional Review Board of the UMCG (local research register number 201800293).

### 2.2. Participants

Patients were included for this study in the case of a malignant pancreatic or periampullary tumor, a routine CPET was performed, underwent pancreatoduodenectomy (including Whipple surgery, pylorus-preserving pancreatoduodenectomy (PPPD) and pylorus-resecting pancreatoduodenectomy (PRPD)), provided informed consent for the FRAIL study, and were 18 years or older. Exclusion criteria were an abdominal CT scan older than 2 months prior to resection, no imaging of level of L3 on CT and incomplete data of immunonutritional parameters.

### 2.3. Body Composition

For this study, the body composition of the patient was assessed using preoperative abdominal CT scans performed as part of standard care. In case of multiple CT scans performed within 2 months prior to resection, the CT scan closest to resection was used. CT imaging was performed on a Siemens Somatom Definition (AS, Flash, Edge), Somatom Force scanner (Siemens Healthineers, Forchheim, Germany) or Biograph128 Vision Quadra Edge (Siemens Medical Solutions USA, Hoffman Estates, IL, USA), GE Medical Systems Optima CT660 (GE Healthcare Japan, Hino, Tokyo, Japan), Toshiba Acquilioin (Prime, Prime SP, One) (Toshiba Medical Systems/Canon Medical Systems, Otawara, Tochigi, Japan), Philips iCT 256 or Philips Ingenuity CT scanner (Philips Medical Systems, Cleveland, OH, USA). All CT images were acquired using an intravenous contrast agent and scanned in the venous phase. At the level of L3, in the slice in which both transverse processes were best visible, skeletal muscle and adipose tissue in cross-sectional CT images were segmented and analyzed using in-house artificial intelligence-assisted analysis software (SarcoMeas AI, version 1.0, UMCG, Groningen, The Netherlands). The segmentations were supervised and where necessary corrected by an experienced radiologist (AV). The skeletal muscle area (SMA) was determined using predefined Hounsfield unit thresholds (−29 to 150 HU) and consisted of the psoas, para-spinous, transversus, rectus abdominis and internal and external oblique muscles. The SMA was used to calculate the skeletal muscle index (SMI) by dividing the SMA by the square of the patient’s height in meters. The SMI (cm^2^/m^2^) was used to quantify sarcopenia. The skeletal muscle density (SMD) was used to quantify myosteatosis and was determined using the average muscle radiodensity, expressed in Hounsfield Units (HUs). No specific cut-off values for BMI and/or sex were used.

### 2.4. Cardiopulmonary Exercise Testing

The aerobic fitness of the patient was assessed using preoperative CPET in case of a potential low aerobic capacity identified by the prehabilitation screening, which was standard care [24,25]. The CPET was performed by a certified sports physician, whereby the ventilatory oxygen uptake (VO_2_) at the ventilatory anaerobic threshold (VAT) and at maximum effort (VO_2peak_) were used as parameters for the aerobic fitness of the patient in this study. When the patient was advised to follow an exercise program, a second CPET was performed to verify whether the aerobic fitness had improved after the exercise program. In these cases, the data from the second CPET was used.

### 2.5. Immunonutritional Status

The preoperative immunonutritional status of the patient was assessed using biomarkers. The lymphocyte-to-monocyte ratio (LMR) (dividing absolute lymphocyte count by monocyte count), neutrophil-to-lymphocyte ratio (NLR) (dividing absolute neutrophil count by lymphocyte count), platelet-to-lymphocyte ratio (PLR) (dividing absolute platelet count by lymphocyte count), CRP-to-albumin ratio (CAR) (dividing CRP in mg/L by albumin in g/L) and prognostic nutritional index (PNI) (10 × albumin in g/dL + 0.005 × absolute lymphocyte count per mm^3^) were used and were all determined by the standard-of-care blood sample at the first outpatient clinic visit prior to surgery. Absolute counts for blood count parameters were in *n* × 10^9^/L.

### 2.6. Other Study Variables

Patient characteristics included age, gender, body mass index (BMI), Charlson Comorbidity Index (CCI) [26], smoking status, alcohol status, ASA-classification [27], and 3-tier risk classification for POPF [28].

### 2.7. Outcomes

The outcome was the association between host factors and failure to achieve TO. TO is defined as the absence of POPF, bile leak, post-pancreatectomy hemorrhage (all grade B/C according to ISGPS or ISGLS), major complications (Clavien–Dindo grade ≥ IIIa), readmission within 30 days after discharge and 30-day mortality [8]. A patient has a TO when all of the above-mentioned conditions are met.

### 2.8. Statistical Analysis

The normality of continuous variables was assessed using histograms and Q-Q plots. Continuous variables were presented as means with standard deviations (SDs) or medians with interquartile ranges [IQR], as appropriate. Between-group differences for continuous variables were analyzed using Mann–Whitney U tests or Student’s *t*-tests based on data distribution. Categorical data were presented as numbers and percentages, with between-group differences evaluated using Chi-square tests, Fisher’s exact tests, or linear-by-linear association as appropriate.

To analyze the relationship between host factors and TO, univariable logistic regression analysis was conducted with 95% confidence intervals (CIs) to calculate odds ratios (ORs) with TO as the outcome. Confounders were also selected based on univariable analysis. Host factors that showed a significant relationship with TO and risk of POPF, defined by the Fistula Risk Score (FRS) [28], since this is a known important confounder of failure to achieve TO, were selected for multivariable backward logistic regression analysis. A *p*-value < 0.05 was considered significant. All data was analyzed using SPSS version 25 (IBM, Armonk, NY, USA).

## 3. Results

### 3.1. Baseline Characteristics

**Figure 1:**
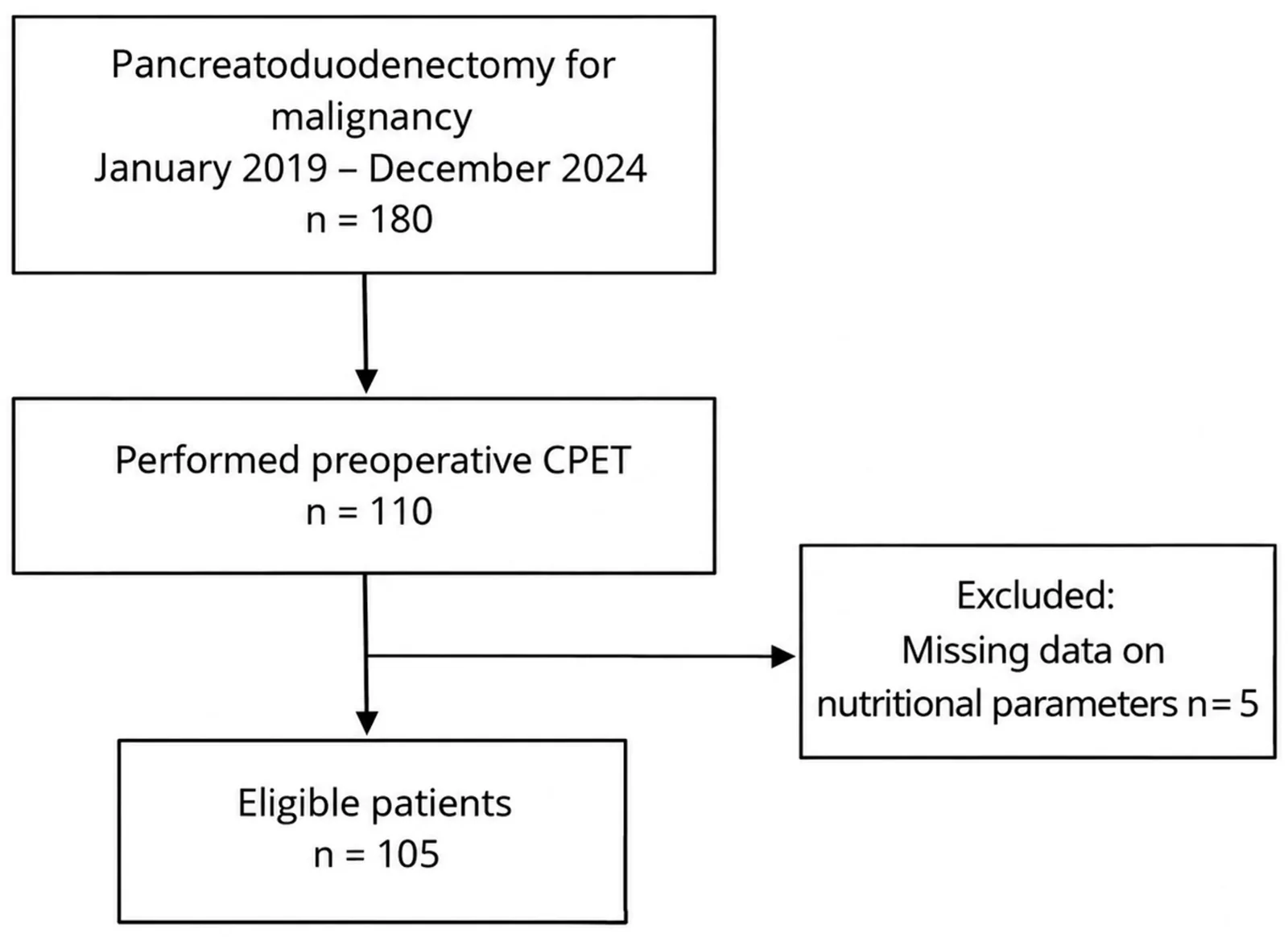
Study flowchart of included patients. CPET = cardiopulmonary exercise test.

This study included 105 patients who underwent a pancreatoduodenectomy for a malignant pancreatic or periampullary tumor between January 2019 and December 2024 (Figure 1). In the total study population, patients had a mean age of 68.3 ± 8.6 years and a mean BMI of 25.6 ± 4.6 kg/m^2^, and 49 patients (46.7%) were female. Patients most often had an ASA-score of II (61.0%) and a median CCI of 3.0 [2.0–4.0].

The median SMI was 41.2 [37.7–46.4] cm^2^/m^2^ and the median SMD was 39.0 [31.9–45.3] HU. Regarding CPET outcomes, the median VO_2peak_ was 20.9 [17.0–23.6] ml/kg/min and VO_2_ at the VAT of 14.3 [12.4–16.2] ml/kg/min. The nutritional parameters had median values of 2.9 [1.9–4.1] for NLR, 175.7 [120.4–246.2] for PLR, 2.6 [1.7–3.4] for LMR, 48.4 [45.7–53.1] for PNI and 1.2 [0.5–3.4] for CAR.

Regarding perioperative characteristics, a PRPD was most often performed (71.4%). All baseline characteristics for the total cohort can be found in Table 1.

### 3.2. Characteristics by TO

In total, 58 patients (55.2%) achieved a TO and 47 patients (44.8%) failed to achieve a TO after pancreatoduodenectomy. To assess host factors that could have an influence on failure to achieve TO, differences in these factors between patients that achieved TO and those who did not (non-TO group) were analyzed (Table 1). Patients in the non-TO group were significantly older than patients in the TO group (70.2 ± 7.8 vs. 66.8 ± 9.0 years).

The PLR was significantly higher in the non-TO group (194.9 [147.3–274.1] vs. 162.8 [105.5–224.6], *p* = 0.016) and the LMR significantly lower in the non-TO group (2.1 [1.4–3.2] vs. 2.9 [1.9–4.2], *p* = 0.005). There were no significant differences in SMI, SMD, VO_2peak_, VO_2_ at the VAT, NLR, PNI and CAR between the groups.

Regarding perioperative risk of developing POPF, patients in the non-TO group were more often classified as high risk (39.1% vs. 22.8%) and the overall classification was significantly different between the groups (*p* = 0.009).

### 3.3. Predictors of Non-TO

With univariable logistic regression analysis, age (OR 1.050, 95%-CI 1.001–1.102, *p* = 0.046), PLR (OR 1.006, 95%-CI 1.001–1.011, *p* = 0.015), LMR (OR 0.599, 95%-CI 0.424–0.845, *p* = 0.004) and risk of POPF category B (OR 3.049, 95%-CI 1.127–8.248, *p* = 0.028) and category C (OR 4.000, 95%-CI 1.413–11.327, *p* = 0.009) showed significant relationships with non-TO (Table 2). After multiple backward logistic regression analysis, only LMR and risk of POPF showed a significant independent relationship with non-TO (Table 2). Patients with a higher LMR had significantly lower odds of failure to achieve TO, with an OR of 0.588 (95%-CI 0.406–0.853, *p* = 0.005). This corresponds with a 1.7-fold higher odds of failure to achieve TO for each 1-unit decrease in LMR (OR 1/0.588 = 1.701, 95%-CI 1.172–2.463). Patients with intermediate or high risk of POPF had significantly higher odds of failure to achieve TO compared to patients with low risk of POPF, with an OR of 3.410 for intermediate risk of POPF (95%-CI 1.184–9.817, *p* = 0.023) and an OR of 3.877 for high risk of POPF (95%-CI 1.310–11.473, *p* = 0.014) compared to the reference (low risk of POPF).

## 4. Discussion

This study investigated objective, preoperatively available, and partially modifiable host factors associated with failure to achieve TO following PD. In our cohort, 44.8% of the patients did not achieve TO. Among all preoperative variables, only the LMR was independently associated with failure to achieve TO after PD. Even after adjusting for POPF risk, lower LMR values significantly increased the likelihood of failure to achieve TO with an OR of 1.701 (95%-CI 1.172–2.463). Neither physical fitness nor body composition parameters demonstrated a statistically significant relationship with failure to achieve TO.

The concept of TO is relatively novel in the field of pancreatic surgery, and widespread use is still limited. Nevertheless, adopting TO offers objective quality assessment of surgical success and aligns closely with the ideal outcome on the patient level. Therefore, the use of TO for assessing surgical outcomes should be widely adopted since it enables more meaningful comparisons across patients and institutions. The overall rate of TO observed in our study (55.2%) is comparable to previously reported rates ranging from 17.5% to 78.3%, with an overall pooled estimate of 44.3% in the literature [10].

In contrast to prior studies, our study did not reveal a relationship between reduced physical fitness and postoperative outcomes. This might be explained by the relatively high fitness levels in our cohort, with a median VO_2_ at the VAT of 14.3 mL/kg/min. A systematic review in hepatobiliary and pancreas cancer surgery showed that a VO_2_ at the VAT below 10.5 mL/kg/min was associated with worse postoperative outcomes [29]. This indicates that only more severely reduced physical fitness is related to adverse postoperative outcomes, while slightly reduced physical fitness, as seen in our population, may not impact the achievement of TO. This is in line with the recent findings by our research group, assessing the relationship between low aerobic fitness and postoperative outcomes. Although there were significantly more patients with delayed gastric emptying in the unfit group, in multivariable regression analysis there was also no significant relation between low aerobic fitness and TO, whereby the fitness level of this cohort was like our cohort [13].

Similarly, no significant association was found between CT-based measures of body composition and TO. Although no previous study investigated the relationship between body composition and TO, there have been studies investigating its relationship with other postoperative complications which contrast with our findings. Van Dijk et al. reported significantly higher rates of surgical site infections in patients with low SMD following pancreatic cancer surgery [30]. Another retrospective study investigated the relationship between low SMI and major complications and showed that patients with low SMI had significantly higher rates of major complications than those with normal SMI [31]. However, these studies only examined individual complications, whereas TO is a more comprehensive, composite outcome. It is therefore possible that while low SMI and low SMD may contribute to specific complications, their impact on achieving TO is more limited or diluted.

The most notable finding in our study is the independent association between a lower preoperative LMR and the likelihood of not achieving TO. LMR is thought to reflect the balance between immune surveillance and pro-tumor inflammation [32] and therefore most existing literature focuses on LMR as a prognostic marker for long-term oncological survival. Evidence regarding its role in short-term surgical outcomes remains limited, but as LMR reflects the inflammatory response it might also be predictive of postoperative outcomes. One retrospective study in distal pancreatectomy found no significant difference in LMR between patients with and without complications [21]. In contrast, in patients with colorectal liver metastasis requiring hepatic resection, a lower median LMR was a predictor of major postoperative complications [33]. In their cohort, PLR was also not significantly associated with postoperative complications, in line with our results. Also outside of oncological surgery, preoperative LMR has been shown to be a predictor of postoperative complications in adhesive small bowel obstruction surgery [34]. Taken together, our results support LMR as a valuable predictor of short-term surgical outcomes in PD, in line with previous evidence from other oncological and non-oncological surgical settings.

In our study, other immunonutritional markers, such as PLR, NLR, CAR and PNI, did not show a significant relationship with TO. These findings align with a recent study of patients undergoing PD for pancreatic cancer, which found no association between NLR or PNI and postoperative complications [35]. However, the literature remains inconsistent, since another study found that PNI and NLR were significant predictors of postoperative complications following PD [22]. This highlights the need for future studies to clarify the role of immunonutritional markers in predicting the postoperative course following PD.

Our findings suggest that immunonutritional status, particularly LMR, may play a valuable role in identifying patients at risk of failure to achieve TO. More importantly, LMR, and the underlying systemic inflammation it reflects, may constitute a modifiable target for preoperative optimization as exercise may limit systemic inflammation through improvement of mitochondrial function [36,37]. Combining LMR with an objective physical fitness assessment (e.g., CPET) could therefore help identify patients who benefit the most from exercise prehabilitation. Nutritional status is closely linked to immune function, so targeted nutritional interventions may potentially reduce systemic inflammation, which could be expressed as an increase in the LMR [38]. Finally, combining objective measures with other modifiable host factors, such as frailty, psychological resilience, comorbidities and intoxications could provide a more holistic approach to the preoperative patient journey and aid in shared decision-making. Future prospective studies should focus on developing and validating such integrated models to improve prediction of surgical outcomes. Our study has several limitations. First, the relatively small sample size may have limited statistical power. Next to this, a data-driven variable selection strategy may result in model instability and may lead to the exclusion of clinically relevant variables. Given the limited number of outcome events, we aimed to restrict the number of variables included in the multivariable model to reduce model complexity and the potential risk of overfitting. Second, the retrospective design may have introduced selection bias and residual confounding. Third, although our study demonstrates an association between LMR and failure to achieve TO, the clinical applicability and an evidence-based optimal threshold for LMR have not yet been established. A prospective study design could mitigate these limitations and allow more extensive data collection to determine such optimal thresholds. Furthermore, potential selection bias related to CPET assessment should be acknowledged. CPET was restricted to patients identified during screening as potentially having low aerobic capacity. Some of these patients underwent exercise intervention as well, and post-intervention CPET measurement was used in this analysis. This methodological approach may have complicated the assessment of baseline physical fitness in relation to postoperative outcomes and could have contributed to the findings for VO_2_-related parameters.

Nonetheless, this study also has important strengths. It is, to the best of our knowledge, the first to evaluate TO in the context of simultaneously available objective data on preoperative aerobic fitness, CT-based body composition, and immunonutritional markers. This integrated approach provides a more complete assessment of host factors. Additionally, the use of TO as an outcome measure allows for a holistic and patient-centered perspective on surgical success. Another strength is that we used CT for body composition assessment, which was recently identified as the gold standard for the assessment of body composition in the updated ESPEN guideline on clinical nutrition in surgery [39]. Lastly, only patients with malignant tumors were included because malignancy is characterized by cancer cachexia, which has an impact on physical fitness, body composition and immunonutritional status [40]. Including patients with benign disease would therefore introduce heterogeneity and confounding, as these patients do not have cancer cachexia and comparison of host factors between benign and malignant tumors would not be valid.

## 5. Conclusions

To our knowledge, this is the first study that investigated the association between objective, potentially modifiable preoperative host factors and TO following PD for pancreatic and periampullary cancer. Our results showed that a lower LMR is independently associated with failure to achieve TO, adjusted for a higher fistula risk score, while no significant associations were found for physical fitness or body composition. These findings suggest that preoperative LMR could potentially be a relevant predictor of failure to achieve TO and a potential target for preoperative optimization. Future prospective research is necessary to validate these results, determine evidence-based cut-off values and explore the integration of multiple objective, partially modifiable, preoperative host factors into comprehensive predictive models.

## Figures and Tables

**Table 1:** Baseline characteristics for the total cohort and by TO group.

	Total Cohort (*n* = 105)	TO (*n* = 58)	Non-TO (*n* = 47)	*p*-Value
**Age (years)**	68.3 ± 8.6	**66.8 ± 9.0**	**70.2 ± 7.8**	**0.043**
Gender, female	49 (46.7)	24 (41.4)	25 (53.2)	0.245
BMI (kg/m^2^)	25.6 ± 4.6	26.0 ± 5.0	25.2 ± 4.0	0.416
ASA-score				0.306
I	5 (4.8)	2 (3.4)	3 (6.4)	
II	64 (61.0)	33 (56.9)	31 (66.0)	
III	35 (33.3)	23 (39.7)	12 (25.5)	
IV	1 (1.0)	0	1 (2.1)	
CCI	3.0 [2.0–4.0]	3.0 [2.0–4.0]	4.0 [3.0–5.0]	0.098
Alcohol use	42 (40.0)	26 (44.8)	16 (34.0)	0.318
Smoking	24 (22.9)	17 (29.3)	7 (14.9)	0.103
Tumor type:				0.449
*PDAC*	49 (46.7%)	31 (53.4%)	18 (38.3%)	
*Duodenal*	6 (5.7%)	3 (5.2%)	3 (6.4%)	
*Bile duct*	31 (29.5%)	13 (22.4%)	18 (38.3%)	
*Ampullary*	19 (18.1%)	11 (19.0%)	8 (17.0%)	
Resection type				0.696
*PRPD*	75 (71.4%)	40 (69.0%)	35 (74.5%)	
*PPPD*	23 (21.9%)	12 (20.7%)	11 (23.4%)	
*Whipple*	7 (6.7%)	6 (10.3%)	1 (2.1%)	
Neo-adjuvant Chemotherapy (Folfirinox)	16 (15.2%)	10 (17.2%)	6 (12.8%)	0.528
Radicality of operation				0.266
R0	66 (62.9%)	33 (56.9%)	33 (70.2%)	
R1	39 (37.1)	25 (43.1%)	14 (29.8)	
Preoperative biliary drainage	74 (70.5%)	39 (67.2%)	35 (74.5%)	0.128
SMI (cm^2^/m^2^)	41.2 [37.7–46.4]	42.2 [38.7–47.4]	39.1 [37.4–45.8]	0.079
SMD (HU)	39.0 [31.9–45.3]	39.2 [33.8–46.1]	36.3 [28.9–43.7]	0.246
VO_2peak_ (mL/kg/min)	20.9 [17.0–23.6]	21.1 [18.2–23.8]	19.9 [15.9–22.7]	0.259
VO_2_ at the VAT (mL/kg/min)	14.3 [12.4–16.2]	14.5 [12.6–16.2]	14.1 [12.4–16.2] *	0.785
NLR	2.9 [1.9–4.1]	2.8 [1.9–3.6]	3.1 [1.8–5.1]	0.152
**PLR**	175.7 [120.4–246.2]	**162.8 [105.5–224.6]**	**194.9 [147.3–274.1]**	**0.016**
**LMR**	2.6 [1.7–3.4]	**2.9 [1.9–4.2]**	**2.1 [1.4–3.2]**	**0.005**
PNI	48.4 [45.7–53.1]	49.4 [46.6–53.4]	47.6 [44.7–52.1]	0.152
CAR	1.2 [0.5–3.4]	0.85 [0.44–2.11]	1.82 [0.59–5.14]	0.139
**FRS**				**0.009**
**Low risk**	35 (34.0)	**26 (45.6)**	**9 (19.6)**	
**Intermediate risk**	37 (35.9)	**18 (31.6)**	**19 (41.3)**	
**High risk**	31 (30.1)	13 (22.8) **	18 (39.1) **	

Values are presented as mean ± SD, median [IQR], or number (%), as appropriate. *p*-values are given for differences between TO and non-TO groups and *p*-values < 0.05 are marked bold. Abbreviations: ASA = American Society of Anesthesiologists; BMI = body mass index; CAR = C-reactive protein to albumin ratio; CCI = Charlson comorbidity index; FRS = fistula risk score; HU = Hounsfield unit; LMR = lymphocyte-to-monocyte ratio; NLR = neutrophil-to-lymphocyte ratio; PLR = platelet-to-lymphocyte ratio; PNI = prognostic nutritional index; PPPD = pylorus-preserving pancreatoduodenectomy; PRPD = pylorus-resecting pancreatoduodenectomy; preoperative biliary drainage consisted of endoscopic retrograde cholangiopancreatography (ERCP) or percutaneous transhepatic biliary drainage (PTBD); SMD = skeletal muscle density; SMI = skeletal muscle index; VAT = ventilatory anaerobic threshold; VO_2_ = ventilatory oxygen uptake. * Two missing. ** One missing.

**Table 2:** Univariable and multivariable logistic regression analysis with non-TO as the dependent variable.

	Univariable Logistic Regression			Multivariable Logistic Regression		
	OR	*p*-Value	95%-CI	OR	*p*-Value	95%-CI
**Age (years)**	**1.050**	**0.046**	**1.001–1.102**	Removed		
BMI (kg/m^2^)	0.965	0.413	0.886–1.051			
CCI	1.271	0.097	0.958–1.687			
SMI (cm^2^/m^2^)	0.959	0.162	0.904–1.017			
SMD (HU)	0.977	0.313	0.933–1.022			
VO_2peak_ (mL/kg/min)	0.975	0.522	0.902–1.054			
VO_2_ at the VAT (mL/kg/min) *	0.993	0.907	0.889–1.110			
NLR	1.171	0.083	0.980–1.400			
**PLR**	**1.006**	**0.015**	**1.001–1.011**	Removed		
**LMR**	**0.599**	**0.004**	**0.424–0.845**	**0.588**	**0.005**	**0.406–0.853**
PNI	0.973	0.364	0.918–1.032			
CAR	1.009	0.798	0.945–1.076			
**FRS**						
**Low risk**	**Reference**			**Reference**		
**Intermediate risk**	**3.049**	**0.028**	**1.127–8.248**	**3.410**	**0.023**	**1.184–9.817**
**High risk**	**4.000**	**0.009**	**1.413–11.327**	**3.877**	**0.014**	**1.310–11.473**

Values are presented as Odds Ratio (OR) and 95% Confidence Interval (CI). *p*-values < 0.05 are marked bold. Terms that were removed during the multivariable backwards logistic regression analysis are indicated with ‘removed’. Abbreviations: BMI = body mass index; CAR = C-reactive protein to albumin ratio; CCI = Charlson comorbidity index; CRP = C-reactive protein; FRS = fistula risk score; HU = Hounsfield unit; LMR = lymphocyte-to-monocyte ratio; NLR = neutrophil-to-lymphocyte ratio; PLR = platelet-to-lymphocyte ratio; PNI = prognostic nutritional index; PPPD = pylorus-preserving pancreatoduodenectomy; PRPD = pylorus-resecting pancreatoduodenectomy; SMD = skeletal muscle density; SMI = skeletal muscle index; VAT = ventilatory anaerobic threshold; VO_2_ = ventilatory oxygen uptake. * Two missing.

## Data Availability

The datasets generated during and/or analyzed during the current study are available from the corresponding author on reasonable request.
